# Characteristics of Child Temperament and Its Association with Mastery Motivation: A Comparative Study in Young Children with and Without Global Developmental Delays

**DOI:** 10.3390/children13050692

**Published:** 2026-05-19

**Authors:** Chien-Lin Lin, Pei-Jung Wang

**Affiliations:** 1 Department of Physical Medicine and Rehabilitation, China Medical University Hospital, Taichung 413305, Taiwan; 005699@tool.caaumed.org.tw; 2 Department of Physical Therapy, Asia University, Taichung 413305, Taiwan

**Keywords:** mastery motivation, temperament, developmental delay, early intervention

## Abstract

**Highlights:**

**What are the main findings?**

• Children with GDD exhibited significantly higher extraversion and lower effortful control compared to children with TD.
• Domain-specific relationships between child temperament and mastery motivation were found in both the GDD and TD groups.

**What are the implications of the main findings?**

• Practitioners should assess a child’s specific temperament trait to design interventions that build upon their natural strengths.
• Targeting the significantly lower effortful control in children with GDD is critical, as improving self-regulation serves as a cornerstone for developing persistence and future developmental competencies.

**Abstract:**

**Background/Objectives:** Child temperament and mastery motivation are key factors for future child competencies. Therefore, two purposes of this study were: (1) to compare temperament characteristics in children with global developmental delay (GDD) and typical development (TD); (2) to explore the relations among child temperament and mastery motivation in GDD and TD groups. This study design was cross-sectional. **Methods**: Participants included 26 children with GDD (ages 2–5) recruited from clinical settings in Central Taichung. A comparison group of 26 TD children, matched for sex and mental age, was subsequently enrolled through community advertisements and local early childhood centers. The participants were invited to our laboratory in order to conduct child development assessment using the Comprehensive Developmental Inventory for Infants and Toddlers, and their mothers were asked to fill in questionnaires, including the revised Dimension of Mastery Questionnaire (DMQ 18) with preschool version to assess mastery motivation and the Child Behavior Questionnaire—Short Form to obtain three dimensions of child temperament (surgency/extraversion, negative affect, effortful control). Correlations and hierarchical regressions were used to examine associations (*p* < 0.05, two-tailed). **Results:** Children with GDD exhibited higher extraversion and lower effortful control than children with TD, whereas no group difference was observed in negative affect. Domain-specific relationships between child temperament and mastery motivation were found in both the GDD and TD groups. **Conclusions:** These findings suggest that early childhood practitioners should consider children’s temperamental and mastery motivation characteristics, identify individual strengths and weaknesses, and design strength-based intervention strategies to promote engagement in daily activities within natural settings.

## 1. Introduction

Early childhood is a foundational period where emerging individual differences significantly predict long-term academic and psychosocial outcomes. Two influential constructs in this trajectory are child temperament and mastery motivation. While temperament represents stable individual traits, recent perspectives suggest these traits are refined through parent–child dyadic regulatory processes [1]. While temperament has traditionally been categorized as a set of stable individual traits (e.g., the CBQ framework), recent perspectives emphasize that these traits emerge and are refined within interpersonal systems. As noted by Christou and Bacopoulou (2025) [2], a child’s ability to manage emotions develops through the biological and cognitive “synchrony” shared with their parent. Because of this, the differences in temperament seen in this study likely reflect how well these shared regulatory processes are functioning within the parent–child pair [2,3,4]. The Children’s Behavior Questionnaire (CBQ) operationalizes temperament into three broad factors: (1) surgency/extraversion: characterized by positive affect, high activity levels, and an approach-oriented nature toward novelty; (2) negative affectivity: reflects the propensity to experience emotions such as fear, anger, and sadness; (3) effortful control: a regulatory dimension involving the ability to inhibit dominant responses, shift attention, and plan actions. This factor is considered a cornerstone of socio-emotional competence [2,5,6,7].

Research on temperament in children with developmental delays remains limited and inconsistent. While some studies suggest children with global developmental delays (GDD) exhibit higher surgency and lower effortful control, others report no significant differences compared to typically developing (TD) peers. Children with Down syndrome often exhibit lower levels of negative affect in infancy, whereas toddlers with autism spectrum disorder (ASD) and boys with Fragile X syndrome frequently display higher negative affect and significant low surgency (social withdrawal) [8,9,10]. Children with ASD showed lower surgency and effortful control and higher negative affectivity than both children with typical development (TD) and GDD [11,12]. Children with global developmental delays (GDDs) showed more surgency and lower effortful control than children with TD, but other studies showed no significant differences in temperament characteristics between children with and without GDD [13,14,15]. These results emphasize the need for syndrome-specific clinical assessments and interventions that account for the unique temperamental strengths and weaknesses of each child. However, it is inconclusive whether temperament characteristics of young children with global delays.

Concurrently, child mastery motivation has been identified as a key factor in later child development. Mastery motivation is defined as a multifaceted, psychological urge that stimulates the individual’s persistent attempts to master tasks that are at least moderately challenging for them personally, even if initial attempts are unsuccessful [16]. There are at least three domains of mastery motivation: cognitive (attempts to solve tasks or problems), social (attempts to master interpersonal relationships with adults and with peers) and gross motor (attempts to master physical skills) [17]. Within each domain, task-directed persistence (a child’s focused and persistent attempt to solve problems or master tasks) and affection (such as Mastery Pleasure and Negative Reactions to Challenges) are used as behavioral indicators of mastery motivation [18]. Understanding the interplay between these two constructs is crucial for promoting optimal development, particularly for children facing developmental challenges. Child temperament is defined as a biologically embedded system of action readiness and emotional reactivity that serves as the foundation for intersubjective communication. It encompasses constitutionally based individual differences in surgency, negative affect, and effortful control, where the latter provides the regulatory scaffolding necessary to transform raw emotional arousal into the consciously experienced, goal-directed persistence known as mastery motivation. Furthermore, temperament is described as the “experiential matrix” from which emotional expression and later mastery motivation emerge, heavily influenced by dyadic synchrony with the caregiver [2,6,7,19].

Surgency/extraversion refers to a positive affect and the tendency to approach new stimuli, and it may drive the instrumental aspects of mastery and pleasure. Negative affectivity refers to fear, sadness, and anger, which can influence persistence levels and reactions to failure. Effortful control provides the regulatory scaffolding, such as attentional flexibility, which is necessary to maintain goal-directed focus during challenges. Rather than a monolithic construct, effortful control functions here as a regulatory mechanism involving attentional flexibility and inhibitory control. By modulating initial emotional reactivity—specifically the frustration associated with task failure—effortful control allows the child to maintain the goal-directed focus necessary for mastery motivation. This mechanism suggests that high mastery motivation is not merely a behavioral outcome of “persistence”, but is predicated on the ability to cognitively override physiological arousal during challenge. Only one previous study showed that surgency/extraversion was positively associated with Gross Motor Persistence, Social Persistence with Adults, Social Persistence with Children, and Mastery Pleasure [20]. Despite the importance of these constructs, few studies have concurrently examined how specific temperament dimensions predict different domains of mastery motivation in children with GDD. Addressing this gap is essential for designing strength-based interventions that promote functional engagement in natural settings.

Children with GDD show significant delay in achieving developmental milestones in two or more of the following domains: cognition, gross/fine motor, speech/language, social, and self-help activities [21]. Clinically significant delays are defined as performance at or below the second percentile, representing a deviation of at least two standard deviations from the normative mean on standardized testing [22,23]. In pediatric clinical practice, the estimated prevalence of global developmental delay (GDD) is approximately 1% to 3%, reflecting a significant portion of the early childhood population requiring specialized intervention [22]. Children with GDD represent a heterogeneous group facing unique challenges that may impact both their temperamental expression and their motivation to engage with their environment. Studies have indicated that children with developmental delays may exhibit less stable temperament profiles over time compared to their peers with TD and are often described by parents as having more temperamental difficulties [24]. Temperamental characteristics may contribute to elevated parenting stress in families of children with developmental delays [25,26]. Given the critical role of temperament and mastery motivation in shaping developmental outcomes, as well as the unique challenges faced by children with GDD, a deeper understanding of these constructs in this population is essential. Although some studies have compared temperament or mastery motivation between children with GDD and those with TD [15,16,17,27], few studies have concurrently examined the complex interplay between specific temperamental dimensions and different domains of mastery motivation both within and across these groups. This gap in the literature limits early childhood practitioners’ ability to design targeted, strength-based interventions that leverage children’s individual profiles to promote engagement and learning.

Thus, this study aimed to: (1) evaluate differences in temperament between children with GDD and mental-age-matched TD controls, and (2) explore the relationship between temperament and mastery motivation across both populations. Hypotheses: It was hypothesized that (1) the GDD group would demonstrate a temperamental signature significantly different from the TD group and that (2) temperament and mastery motivation would be significantly associated in both developmental contexts.

## 2. Materials and Methods

### 2.1. Study Design

We conducted a case–control design study at a university laboratory in Taiwan. The case–control design, a methodology used to compare individuals with a specific condition or outcome to those without it (the controls) to identify differences in characteristics or exposures. This design is particularly effective in clinical and developmental research for investigating the profiles of low-prevalence populations, such as children with global developmental delays (GDD), relative to typically developing (TD) peers. By matching controls to cases on key variables—in this instance, sex and mental age—the design minimizes the influence of confounding factors, allowing for a more precise examination of the associations between temperament and mastery motivation.

### 2.2. Participants

Seventy-one participants signed the consent form in our study. Young children with GDD were recruited from clinics or day care centers in the greater Taichung area. The inclusion criteria are as follows: (1) children aged between 24 and 60 months; (2) children receiving a doctor’s diagnosis related to GDD (e.g., developmental delay in at least 2 domains); (3) the rater should be the primary caregiver who takes care of the child for at least 4 h per day; and (4) caregiver’s educational level is at least junior high school. The exclusion criteria are as follows: (1) progressive diseases (e.g., neuromuscular dystrophy, brain tumor, etc.; (2) autism spectrum disorder or attention deficit–hyperactivity disorder; (3) children with unstable medical conditions (e.g., epilepsy), severe heart disease (e.g., Tetralogy of Fallot), frequent hospitalization, or receiving a surgical operation in the past 6 months; and/or (4) children’s visual or hearing impairments being severe enough to interfere with test performance despite the use of corrective assistive devices. For every child in the GDD group, a sex- and mental-age-matched typically developing (TD) peer was recruited through clinical referrals, community advertisements, daycare centers, and kindergartens.

We calculated the mental age of each child with GDD, and recruited one child with TD whose chronological age was within 2 months of the GDD child’s mental age and was the same gender. Inclusion criteria for children who were TD are as follows: (1) developmental quotient on the CDIIT was 85 or above, (2) the primary caregiver should take care of the child for at least 4 h daily, and (3) the caregiver’s educational level is at least junior high school.

Initial recruitment included nine children with GDD who were subsequently excluded for not meeting the inclusion criteria, while two mother–child dyads withdrew during the laboratory observation phase. Of the 60 dyads who completed data collection, eight were excluded from final analysis based on pre-defined criteria: five children presented with delays in only a single developmental domain, and one mother provided fewer than four hours of daily primary care. Additionally, two cases were excluded due to extreme response bias on the DMQ, one characterized by consistent ceiling effects and the other by consistent floor effects. Consequently, the final analytical sample comprised 52 dyads, evenly divided between the GDD and TD groups (n = 26 per group).

### 2.3. Measures

#### 2.3.1. Child Behavioral Questionnaire with Short Form

The Child Behavior Questionnaire—Short Form (CBQ-SF) is a widely used parent-report measure of temperament in early childhood [28,29]. The CBQ-SF consists of 94 items across 15 temperament scales. Parents rate each item on a 7-point Likert scale, ranging from 1 (never) to 7 (always) [27,28,29]. The short form was developed based on the factor structure identified in the standard CBQ. Factor analyses of the CBQ-SF have identified three higher-order temperament factors: surgency/extraversion, negative affectivity, and effortful control [28,29].

Surgency/extraversion reflects activity level, impulsivity, high-intensity pleasure, positive anticipation, and sociability. Negative affectivity includes fear, frustration, sadness, and discomfort. Effortful control reflects attentional focusing, inhibitory control, and emerging regulatory capacities. Each higher-order factor score is calculated as the average of its corresponding temperament scales. Previous studies have reported acceptable to good reliability and validity for the CBQ-SF [27,28,29]. In the present study, we used three composite scores representing the higher-order temperament dimensions to characterize children’s temperament.

#### 2.3.2. The Revised Dimensions of Mastery Questionnaire

The Dimensions of Mastery Questionnaire (DMQ 18) is a widely used global instrument for measuring mastery motivation. It utilizes informant ratings from adults familiar with the child—such as parents, caregivers, or teachers—to assess the child’s motivational characteristics [17]. The DMQ 18 comprises six scales: Cognitive Persistence, Gross Motor Persistence, Social Persistence (with Adults and Children), Mastery Pleasure, and Negative Reactions to Challenges. Each item utilizes a five-point Likert response format (1 = not at all like this child; 5 = exactly like this child). Scores are coded such that higher values indicate more robust mastery motivation [17]. The reliability coefficients for the persistence and affective subscales varied from marginal to good, confirming the psychometric adequacy of the DMQ 18 for assessing mastery motivation in this population [16]. The psychometric robustness of the DMQ 18 is well-documented, with numerous studies providing evidence for its strong convergent and construct validity across diverse pediatric populations [30,31,32]. In this study, we used six scale scores (Cognitive Persistence, Gross Motor Persistence, Social Persistence with Adults, and Social Persistence with Children; Mastery Pleasure, and Negative Reactions to Challenges) as indicators of mastery motivation.

#### 2.3.3. Comprehensive Developmental Inventory for Infants and Toddlers (CDIIT)

The CDIIT is a diagnostic developmental test that was standardized on a norm sample of 3703 Taiwanese children, aged 3–72 months; it has six developmental subtests (cognition, language, gross motor, fine motor, social, and self-help). The cognitive subtest is used to assess a child’s mental capacity; the language subtest consists of comprehension and expression. The gross motor subtest includes items to assess antigravity, locomotion and body-movement coordination, and the fine motor subtest includes items for basic hand use and visual–motor coordination. The social subtest is used to assess social interaction, and self-help contains items dealing with feeding, dressing and hygiene skills [33]. The tester administers all the cognitive and motor items and some items of the language subtests; other items of the language subtest, plus the social and self-help subtests, are reported by the main caregiver.

Items are scored dichotomously (0 or 1); a score of 1 is assigned if success is demonstrated during direct assessment or confirmed via caregiver observation. Developmental ages and developmental quotients for all subtests and the whole test were obtained according to Taiwanese norms [33]. A developmental quotient (DQ) less than 70 (2 SD below the mean) on a subtest indicates developmental delay in this study. For children developing typically, their whole DQ from the CDIIT above 85 indicates TD. It takes about 35–40 min to administer CDIIT. The CDIIT has acceptable psychometric properties, including test–retest reliability, construct validity [32], and concurrent validity [34]. In this study, the cognitive and whole DQ were used to assess children’s cognitive abilities and overall developmental abilities, respectively.

### 2.4. Study Procedure

This study was approved by the Human Subjects Review Committee of the participating medical center. Mother–child dyads were invited to attend a 90 min laboratory session. Upon arrival, a warm-up period was provided using engaging toys unrelated to the testing materials to help the child acclimate to the environment. Following a 5 min break, the Comprehensive Developmental Inventory for Infants and Toddlers (CDIIT) was administered by a trained pediatric physical therapist.

Each child’s mental age was calculated immediately after completing the CDIIT and was used to determine an initially appropriate difficulty level for the subsequent mastery tasks. After an additional 5 min break, the child participated in an individualized structured mastery task assessment (data from this procedure were used in a separate study). During this time, the mother completed a demographic questionnaire (including maternal education, family income, and socioeconomic status indexed by paternal education and occupation) and rated the temperament and motivation measures (CBQ-SF and DMQ 18). Mothers completed the questionnaires in the same room, seated with their backs facing the child to minimize potential influence on task performance.

### 2.5. Data Reduction and Analysis

All the outcome variables, temperament, mastery motivation variables, and DQs were examined for normality and statistically analyzed, using IBM SPSS software (version 25) [35]. The main dependent variables were 6 indicators of mastery motivation: Cognitive Persistence, Gross Motor Persistence, Social Persistence with Child, Social Persistence with Peers, Mastery Pleasure, and Negative Reaction. The surgency/extraversion, negative affectivity, and effortful control were measured by mothers’ ratings on the CBQ-SF to obtain three dimensions of temperament variables. The child’s cognitive DQ scores were used to indicate the child’s cognitive ability. All outcome variables, temperament and mastery motivation indicators, and developmental quotients were examined for normality. For the hierarchical regression models, we assessed for multicollinearity among the temperament dimensions; all Variance Inflation Factors (VIFs) were <2.5, indicating that the predictors were sufficiently independent. Additionally, an inspection of residual plots confirmed that the assumptions of normality and homoscedasticity were met.

Maternal education was assessed using a 7-point ordinal scale: 1 (illiterate), 2 (primary school), 3 (junior high school), 4 (senior high school), 5 (junior college), 6 (bachelor’s degree), and 7 (postgraduate degree). This scale served as the maternal education score for analysis. Annual family income was treated as a dichotomous variable, categorized into two levels: 1 (<100,000 NTD) and 2 (≥100,000 NTD). Family socioeconomic status (SES) was determined by paternal education and occupation, with levels ranging from I (highest) to IV [36]. Descriptive statistics were used to present basic information about the children, their families, and the scores of various measures (Table 1). Data collection was conducted by the authors, who are trained in child development. To ensure consistency, all assessments were performed in a standardized clinical setting following a protocol approved by the Institutional Review Board.

For comparison of the differences between the two groups, independent *t*-tests were used for continuous variables with normal distribution. For independent *t*-tests, effect sizes (ES) were calculated as d = mean difference in two groups/standard deviation of the differences [37]. Repeated measure ANOVA and paired *t*-tests were used to examine the within-group differences in three temperament dimensions in the GDD or TD group (significance level: α < 0.05, two-tailed). The correlations of children’s temperament and mastery motivation were analyzed by using Pearson correlations. To further examine the group differences, we used Fisher’s r-to-z transformation analysis to assess the significance of the difference between correlation coefficients of GDD and TD groups.

The hierarchical two-step regression models were conducted to examine the predictive effects of three temperament dimensions for 6 MM indicators (Cognitive Persistence, Gross Motor Persistence, Social Persistence with Adults, Social Persistence with Children, Mastery Pleasure, and Negative Reactions to Challenges), respectively, after considering the contribution of child age and cognitive DQ. Three temperament dimensions (surgency/extraversion, negative affectivity, and effortful control) indicated child temperament characteristics (significance level: α < 0.05, two-tailed).

## 3. Results

### 3.1. Group Characteristics

Table 1 presents the descriptive data of children with GDD and with TD and their families. The proportion of sex did not differ between groups (*p* = 0.10). In addition to GDD, 13 children had other medical diagnoses, including Down syndrome (n = 6), Williams syndrome (n = 1), microcephalus (n = 1), and genetic disorders (n = 5). Although there were significant differences in DQs between the two groups, there were no differences in developmental age on any of the domains of the CDIIT, except the language and social domain (Table 1). As expected, children with GDD demonstrated substantially lower developmental quotients on the CDIIT. Significant group differences were observed for whole DQ and cognitive DQ, with the TD group scoring higher in both domains (all *p* < 0.001).

Regarding mastery motivation, children with GDD showed lower levels of Cognitive/Object Persistence, Gross Motor Persistence, Social Persistence with Adults, Mastery Pleasure, and marginally lower Social Persistence with Children compared with TD peers (*p* values = 0.001–0.05). No group difference was found for Negative Reactions to Challenges (*p* = 0.85).

There were no group differences in family variables (i.e., maternal age, socioeconomic status, annual household income, and maternal education). Most of the mothers in the GDD and TD groups (n = 34, 69%) had earned a college or graduate degree. Most families in the current study were classified as middle-to-high SES.

### 3.2. Group Comparisons of Child Temperament Characteristics

The results of comparisons between the global delay and typical groups on the child temperament dimensions are shown in Table 2. Children with GDD exhibited higher levels of extraversion compared to their TD counterparts (*p* < 0.05, d = 0.58). Conversely, effortful control was significantly lower in the GDD group (*p* < 0.001, d = 1.17), representing a large effect size. No group difference was observed for negative affectivity.

Regarding the three domains of temperament in the GDD group, significant differences were found [F (1,25) = 13.64, *p* = 0.001, η^2^ = 0.94]. We further used a paired *t*-test to make pairwise comparisons, which revealed that the surgency/extraversion scores were significantly lower than negative affectivity or effortful control scores. There was no significant difference between negative affectivity or effortful control scores. For the TD group, there were significant differences [F (1,25) = 13.72, *p* = 0.001, η^2^ = 0.95]. Pairwise comparisons using paired *t*-tests indicated that the effortful control scores were significantly higher than both surgency/extraversion or negative affectivity scores. No significant difference was found between surgency/extraversion and negative affectivity scores.

### 3.3. Associations Between Child Temperament and Mastery Motivation

The results revealed that domain-specific relationships between child temperament and mastery motivation in both groups (Table 3). In the GDD group, surgency/extraversion was positively associated with Gross Motor Persistence, Social Persistence with Adults, Social Persistence with Children, and Negative Reactions to Challenges (*r* = 0.41–0.57, *p* < 0.05). Negative affectivity was positively associated with Gross Motor Persistence, Social Persistence with Children, and Negative Reactions to Challenges (*r* = 0.43–0.60, *p* < 0.05). Effortful control was positively related to Mastery Pleasure (*r* = 0.40, *p* < 0.05).

In the TD group, fewer significant associations were observed (Table 3). Surgency/extraversion was not significantly related to mastery motivation indicators. Negative affectivity showed a moderate positive association with Social Persistence with Children (*r* = 0.47, *p* < 0.05). Effortful control was positively associated with Mastery Pleasure (*r* = 0.39, *p* < 0.05). Comparisons of the correlation coefficients between the two groups indicated no significant group differences in the magnitude of the correlations (z = 0.48–1.03, *p* = 0.21–0.97). These findings suggest that the associations between temperament and mastery motivation were generally similar across the two groups.

Two-step hierarchical regression analyses were conducted to examine the predictive effects of the three temperament dimensions on mastery motivation in children with GDD after controlling for child age and cognitive ability (Table 4). The temperament models explained 44%, 24%, 50%, 19%, and 44% of the variance in Gross Motor Persistence, Social Persistence with Adults, Social Persistence with Children, Mastery Pleasure, and Negative Reactions to Challenges, respectively (*ps* < 0.05). Surgency/extraversion showed significant positive predictive effects on Gross Motor Persistence, Social Persistence with Adults, and Social Persistence with Children (*β* = 0.45–0.71, *ps* < 0.05). Negative affectivity uniquely predicted Negative Reactions to Challenges (*β* = 0.38, *p* < 0.05). Effortful control significantly predicted Gross Motor Persistence, Social Persistence with Adults, Social Persistence with Children, and Mastery Pleasure (*β* = 0.35–0.52, *ps* < 0.05). In contrast, for the TD group, none of the temperament dimensions significantly predicted mastery motivation indicators after controlling for child age and cognitive ability.

## 4. Discussion

The key findings of this study were that children with GDD demonstrated significantly lower effortful control than TD children, with a large effect size, while no group difference was found in negative affectivity. Interestingly, children with GDD showed higher levels of surgency/extraversion compared with their TD peers. Within-group comparisons further indicated different temperament profiles between groups: children with GDD showed relatively lower surgency/extraversion compared with their other temperament dimensions, whereas TD children demonstrated the highest levels of effortful control.

Second, temperament showed domain-specific predictions with mastery motivation in the GDD group even after controlling for child age and cognitive ability. Surgency/extraversion predicted persistence in both gross motor and social contexts, negative affectivity predicted Negative Reactions to Challenges, and effortful control predicted gross motor and social persistence and Mastery Pleasure. However, no significant predictive effects of temperament on mastery motivation were observed in the TD group. Possible reasons for the key findings are discussed below, along with clinical implications.

### 4.1. Group Differences in Temperament

One of the key findings was that young children with GDD demonstrated significantly lower levels of effortful control than their TD counterparts, whereas no significant group difference was found for negative affectivity. In addition, children with GDD showed higher levels of surgency/extraversion compared with TD children. Thus, these findings suggest that differences in self-regulatory capacity may represent a key temperamental characteristic distinguishing children with GDD from typically developing peers. Our findings partially supported our first hypothesis and revealed distinct group differences in specific temperament dimensions. High surgency in GDD may represent undifferentiated action readiness (emotion) that lacks the top-down cortical brake of effortful control. The lower effortful control observed in the GDD group is consistent with theoretical perspectives emphasizing the role of self-regulation in early development. Effortful control involves the ability to regulate attention, inhibit dominant responses, and flexibly engage in goal-directed behavior [19] and previous studies [13,14,15]. Children with GDD often experience delays in cognitive and executive functioning, which may contribute to reduced regulatory capacity and difficulties in maintaining attention or inhibiting impulsive responses [8,38]. Such differences in effortful control may influence children’s engagement in learning activities and their ability to persist when encountering challenges.

Interestingly, children with GDD also demonstrated higher levels of surgency/extraversion than TD children. Surgency/extraversion reflects activity level, approach behaviors toward novelty, and positive affect [28]. Higher levels of surgency may reflect greater activity or impulsivity among children with developmental delays, possibly associated with less developed regulatory processes. Previous studies examining temperament in children with developmental delays have reported mixed findings, with some studies indicating greater surgency or activity levels in children with global delays and others reporting no significant differences between children with and without developmental delays [13,14,15]. The present findings suggested that specific temperament dimensions, particularly effortful control, may differentiate children with GDD from TD peers when groups are matched on mental age.

Within-group analyses further revealed distinct temperament profiles across the three dimensions. In the TD group, effortful control was significantly higher than both surgency/extraversion and negative affectivity, reflecting the increasing development of self-regulatory abilities during the preschool years. In contrast, children with GDD showed relatively lower surgency/extraversion compared with their other temperament dimensions. Thus, these findings suggest that children with GDD and TD children may exhibit different temperamental patterns during early childhood. Understanding these differences may help practitioners better interpret children’s behavioral styles and design interventions that align with individual temperamental characteristics to support engagement and learning [39].

### 4.2. Associations Between Temperament and Mastery Motivation

Another key finding in this study was that temperament showed domain-specific associations with mastery motivation, particularly in the GDD group. In children with GDD, surgency/extraversion was positively associated with several indicators of mastery motivation, including Gross Motor Persistence and Social Persistence with both adults and peers. This finding supports our second hypothesis and aligns with results from two previous studies of young children [20,40]. The above finding has indicated that children who display higher levels of approach behaviors, activity, and positive affect may be more likely to actively engage in challenging tasks and social interactions. Link this to the “Intersubjective Communication” theme. Higher surgency (approach behaviors) serves as the “implicit” engine for social synchrony, which then builds the child’s persistence in social domains. Such characteristics may facilitate children’s willingness to explore their environment and persist in goal-directed activities, which are important components of mastery motivation.

Negative affectivity was also positively associated with several indicators of mastery motivation in the GDD group, including persistence in gross motor tasks and social interactions with children, as well as Negative Reactions to Challenges. Although negative affectivity is often viewed as a risk factor for emotional difficulties, it may also reflect heightened emotional engagement when children encounter challenging situations. In this context, children who show stronger emotional reactions may also demonstrate greater behavioral persistence when attempting to overcome task difficulties. Furthermore, effortful control was positively associated with Mastery Pleasure in the GDD group, suggesting that children with stronger regulation of behavior and attention may experience greater positive affect and satisfaction when successfully mastering challenging tasks. Our findings partially supported the idea that children’s self-regulation has a positive association with mastery motivation [41].

In contrast, fewer significant associations between temperament and mastery motivation were observed in the TD group. Surgency/extraversion was not significantly related to mastery motivation indicators, while negative affectivity showed only a moderate association with social persistence with peers. Effortful control was positively related to Mastery Pleasure, consistent with the role of self-regulation in supporting positive emotional responses during task mastery [41]. Despite these differences in the number of significant associations, comparisons of correlation coefficients indicated that the magnitude of the relationships between temperament and mastery motivation did not significantly differ between the two groups. Overall, these findings suggest that temperament characteristics may play a more observable role in shaping mastery-related behaviors among children with GDD, although the underlying relationships between temperament and motivation appear to be broadly similar across developmental groups.

### 4.3. Implications

The present findings highlight several important clinical implications for early intervention practice with young children with GDD. First, the lower levels of effortful control observed in children with GDD suggest potential difficulties in self-regulation, attention control, and inhibitory processes. These challenges may influence children’s persistence in challenging tasks and their ability to regulate emotional responses when facing obstacles. Practitioners may therefore benefit from incorporating strategies that support self-regulation and task engagement, such as structured routines, clear expectations, and appropriate scaffolding during learning activities. Understanding children’s temperament may also help practitioners determine appropriate task demands, instructional formats, and environmental structures to support successful participation [39].

Second, the findings indicate domain-specific relations between temperament characteristics and mastery motivation, particularly among children with GDD. Understanding a child’s temperament profile may help practitioners tailor intervention and educational strategies to enhance children’s engagement and persistence in therapeutic and learning tasks [39]. For example, practitioners should use attentional synchrony (e.g., joint gaze and exaggerated facial expressions) to “catch” the child’s high energy and pivot it toward a goal before the child’s attention shifts [2]. Also, caregivers should provide contingent affect labeling (e.g., “I see you are frustrated because this is hard”). This linguistic scaffolding helps the child transform a diffuse, distressing bodily state into a consciously represented feeling—the first step toward self-regulation [2]. Furthermore, practitioners should incorporate rhythmic, synchronous activities (e.g., drumming together, breathing exercises, or mirrored movements) to enhance autonomic and neural coupling between the child and practitioners.

Finally, considering child temperament may also support family-centered practice. Children’s temperament can influence parental perceptions, parent–child interactions, and parenting stress in families of children with developmental disabilities [4,39]. Furthermore, we should frame intervention as “dyadic scaffolding.” It suggests that practitioners act as the child’s “external prefrontal cortex” to help translate their high surgency/reactivity into successful task completion (mastery). Thus, helping parents understand that some behavioral tendencies may be biologically influenced rather than intentional misbehavior may encourage more supportive responses and effective strategies for managing challenging situations.

Overall, these findings underscore the importance of adopting a strength-based and individualized approach in early intervention. By recognizing and leveraging children’s temperamental strengths, practitioners can design more engaging intervention environments that promote persistence, mastery, and overall developmental progress.

### 4.4. Limitations

There were several limitations, such as the following: (1) our study design was a case–control study, and a relative lack of longitudinal research on the temperament profiles of young children with disabilities; (2) reliance on parent-report measures for both temperament and mastery motivation. This creates the potential for shared method variance, which may inflate the observed associations between these constructs. Furthermore, since the caregiver is the “scaffold” for the child’s emerging feelings and self-regulation, their report reflects a dyadic perception of the child’s behavior within their specific relational context. Future research should employ multi-method approaches, such as eye-tracking for attentional biases or fNIRS for neural synchrony, to provide objective markers that complement caregiver insights; (3) the relatively small sample size (n = 52 dyads) may limit the statistical power of the hierarchical regression models and the generalizability of the findings. While the large effect sizes observed in temperament differences between groups suggest robust findings, the complexity of the interplay between biologically embedded action readiness (temperament) and consciously represented feelings (mastery motivation) may require larger cohorts to fully elucidate. Consequently, these results should be interpreted with caution and viewed as a foundation for future large-scale, multi-center investigations [15].

### 4.5. Recommendations for Future Research

Based on the limitations of the current study, several avenues for future research are recommended to further clarify the relationship between temperament and mastery motivation in clinical populations. First, as this study employed a cross-sectional case–control design, future research should utilize longitudinal designs. Tracking children with GDD over extended periods would allow researchers to observe the stability of temperamental profiles and determine the long-term developmental trajectories of mastery motivation. Such studies are essential for establishing the causal nature of the associations identified here. Second, future investigations should adopt a multi-method assessment approach. While parental reports provide valuable ecological insight into a child’s behavior, they may be subject to subjective bias. Incorporating direct behavioral observations and laboratory-based tasks alongside caregiver reports would provide a more objective and comprehensive understanding of the underlying mechanisms driving these traits. Finally, future studies should aim for larger and more diverse samples. Increasing the sample size would provide the statistical power necessary for more complex multivariate analyses, such as structural equation modeling. Furthermore, expanding recruitment to include families from varied socioeconomic backgrounds and diverse ethnic regions beyond the current focus on middle-to-upper SES Asian populations would enhance the generalizability of the findings. Research involving broader demographic groups is necessary to determine if these temperamental and motivational patterns are universal or culturally specific.

## 5. Conclusions

In this study, young children with GDD showed lower effortful control and higher surgency/extraversion than their TD peers, while no group differences were observed in negative affectivity. The low effortful control in GDD children, described as a disruption in the developmental bridge from raw emotional reactivity to organized, is reflective of feelings of mastery. Furthermore, temperament showed domain-specific associations with mastery motivation, particularly among children with GDD. Surgency/extraversion and effortful control were associated with persistence and Mastery Pleasure, whereas negative affectivity was related to children’s Negative Reactions to Challenges. In contrast, temperament did not significantly predict mastery motivation indicators in the TD group. These findings highlight the importance of considering temperament characteristics when understanding motivational behaviors in young children with developmental delays. Therefore, it is crucial for practitioners to assess children’s temperament profiles and design individualized strategies that also educate parents to promote persistence and engagement in daily activities.

## Figures and Tables

**Table 1:** Characteristics of the children with global developmental delay and with typical development (n = 26 for each group).

Variables	Global Delays	Typical Development	*p* ^a^
Child Variables			
Age (Months) ^a^	41.2 (9.4)	32.2 (10.4)	<0.001
Male/Female (n, %)	17 (65%)/9 (35%)	11 (42%)/15 (58%)	0.10
DQ of CDIIT ^a^			
Whole DQ	59.8 (8.2)	99.2 (10.9)	<0.001
Cognitive DQ	61.1 (9.3)	94.4 (10.1)	<0.001
Mastery Motivation of DMQ 18			
Cognitive/Object Persistence	3.02 (0.81)	3.52 (0.74)	0.02
Gross Motor Persistence	3.21 (0.91)	3.97(0.62)	0.001
Social Persistence with Adults	3.19 (0.88)	3.96 (0.61)	0.001
Social Persistence with Children	2.97 (1.05)	3.45 (0.62)	0.05
Mastery Pleasure	4.14 (0.82)	4.57 (0.39)	0.02
Negative Reactions to Challenges	3.45 (0.85)	3.49 (0.58)	0.85
Family Variables			
Caregivers’ Age (Years) ^a^	35.6 (5.2)	34.0 (4.1)	0.12
Caregivers’ Education Level (n, %) ^b^			
≧college	24, 92%	18, 69%	0.06

*Note*: ^a^ Independent *t*-test (two-tailed). ^b^ Chi-squared test. All measurements are expressed as mean (SD). Abbreviations: CDIIT = The Comprehensive Developmental Inventory for Infants and Toddlers, and DQ = developmental quotient.

**Table 2:** Comparison of mental age-matched children with and without global developmental delay (GDD) on the subscales of the CBQ scales.

CBQ Scales	GDD (n = 26) M (SD)	TD (n = 26) M (SD)	*t* ^a^	*p* ^a^	*d*
Surgency/extraversion (surgency)	4.79 (0.94)	4.32 (0.64)	2.10	0.04	0.58
Negative affectivity (NA)	4.12 (0.96)	4.08 (0.68)	0.18	0.86	0.24
Effortful control (EC)	3.87 (0.75)	4.67 (0.60)	−4.21	<0.001	1.17
Within-group effect ^b^	Surgency > NA~EC ***	EC > Surgency~NA ***			

*Note*: ^a^ Independent *t*-test (two-tailed) for between-group effect. ^b^ Paired-*t*-test for within-group effect. All measurements are expressed as mean (SD). Abbreviations: GDD = global developmental delay; CBQ-short form = the Child Behavioral Questionnaire—Short Form. *** *p* < 0.05.

**Table 3:** Associations between temperament and mastery motivation in children with global developmental delay and with typical development.

Variables	Surgency/Extraversion		Negative Affectivity		Effortful Control	
n = 26 for each group	GDD	TD	GDD	TD	GDD	TD
Cognitive/Object P	0.06	0.01	0.31	0.19	0.37	0.37
Gross Motor P	0.55 **	0.19	0.43 *	0.05	0.25	0.19
Social P with Adult	0.41 *	0.37	0.36	0.31	0.33	−0.02
Social P with Children	0.57 **	0.33	0.60 **	0.47 *	0.36	0.17
Mastery Pleasure	0.33	−0.12	0.32	0.14	0.40 *	0.39 *
Negative Reactions to Challenges	0.41 *	0.31	0.56 **	0.35	0.23	0.00

* *p* < 0.05; ** *p* < 0.01.

**Table 4:** Predicting the effect of child temperament on mastery motivation in young children with global developmental delays.

Outcome Variables	COP		GMP		SPA		SPC		MP		NRC	
Predictors	*B*	*β*	*B*	*β*	*B*	*β*	*B*	*β*	*B*	*β*	*B*	*β*
	Adjusted R^2^ = 0.12 (F = 1.67)		Adjusted R^2^ = 0.44 ** (F = 4.87 **)		Adjusted R^2^ = 0.24 ^#^ (F = 2.58 ^#^)		Adjusted R^2^ = 0.50 ** (F = 6.03 **)		Adjusted R^2^ = 0.19 * (F = 2.16 *)		Adjusted R^2^ = 0.44 ** (F = 4.93 **)	
**Step 1**												
**Child Age**	0.02	0.18	0.01	0.12	0.03	0.28	0.05	0.42 *	0.02	0.26	0.03 ^#^	0.35 ^#^
**CDQ**	0.02	0.26	0.02	0.21	0.02	0.22	0.02	0.17	0.01	0.14	0.04	0.41 *
**Step 2**												
**Child Age**	−0.002	−0.02	−0.03	−0.33	−0.01	−0.06	0.01	0.06	−0.01	−0.08	0.01	0.11
**CDQ**	0.03	0.33	0.03	0.29	0.03	0.30	0.03	0.24	0.02	0.23	0.04 *	0.45 *
**Surgency**	−0.01	−0.02	0.68 **	0.71 **	0.43 *	0.47 *	0.49 *	0.45 *	0.36 ^#^	0.42 ^#^	0.17	0.18
**NA**	0.19	0.23	0.02	0.02	0.01	0.01	0.29	0.26	−0.02	−0.02	0.34 *	0.38 *
**EC**	0.410	0.38	0.60 *	0.50 *	0.53 *	0.45 *	0.49 *	0.35 *	0.57 *	0.52 *	0.22	0.19

*Note.* * *p* < 0.05; ** *p* < 0.01; and ^#^ *p* = 0.05–0.1 (two-tailed). By hierarchical regression. Significant results are in bold type. Abbreviation: β = standardized regression coefficient; B = unstandardized regression coefficient; CDQ = cognitive developmental quotient; COP = Cognitive Persistence; GMP = Gross Motor Persistence; SPA = Social Persistence with Adults; SPC = Social Persistence with Children; MP = Mastery Pleasure; and NRC = Negative Reactions to Challenges.

## Data Availability

Data are not publicly available due to privacy and ethical restrictions.
